# Morel-Lavallée Lesion

**Published:** 2014-04-25

**Authors:** Jonathan Miller, Justin Daggett, Raj Ambay, Wyatt G. Payne

**Affiliations:** ^a^Division of Plastic Surgery, Department of Surgery, University of South Florida College of Medicine, Tampa, Fla; ^b^Plastic Surgery Section, James A. Haley VA Healthcare System, Tampa, Fla; ^c^Plastic Surgery Section, Bay Pines VA Healthcare System, Bay Pines, Fla

**Keywords:** Morel-Lavallée, seroma, traumatic hematoma, degloving injury, shearing injury

## DESCRIPTION

A 60-year-old man presented with an expanding mass of the left trochanter/lower back. The patient was struck by a motor vehicle 4 weeks prior and sustained nonoperative pelvic and L5 fractures. Following the accident, a hematoma was noted over the left trochanter, which was initially managed conservatively.

## QUESTIONS

1. **What is the mechanism behind a Morel-Lavallée (ML) lesion?**
2. **What is the differential diagnosis for soft tissue swelling in the greater trochanter area and where else may an ML lesion present?**
3. **What diagnostic imaging should be considered?**
4. **What treatment options are available for this condition?**

## DISCUSSION

Morel-Lavallée lesions are a form of closed degloving injury that leads to fluid accumulation and the formation of a pseudocyst.^1^ Morel-Lavallée lesions are most often found over the greater trochanter and may also be seen over the knee, lumbar region, or scapula.^1^^,^^2^ Following a closed shearing force injury, it is possible for the subcutaneous fat to be separated from the deep fascia. Within this space, blood, serous fluid, and other materials may become trapped.

Patients with ML lesions often present with soft tissue swelling, bulging, bruising, and decreased cutaneous sensation in the affected area.^1^ Although fairly distinct in their presentation, the differential diagnosis for such lesion may include sarcomas, hemangiomas, aneurysmal bone cyst, and subcutaneous hematomas.^3^

Ultrasonographic imaging may show a thickened capsule surrounding either hypoechoic or anechoic area. Furthermore, echogenic foci may be seen depending on the amount of debris within the space.^4^ Magnetic resonance imaging remains the preferred imaging technique for its ability to visualize soft tissue. Morel-Lavallée lesions will appear as a discrete collection of fluid between the subcutaneous fat and underlying fascia. Similar to ultrasonographic imaging, fat and debris may be visualized as well as fluid-fluid levels.^3^

Depending on how early the ML lesion is diagnosed, conservative therapy may be enough in its management. If the lesions are within the trochanter, pelvic girdle, or gluteal region, compression bandaging alone can lead to resolution in an average of around 6 weeks.^5^ Furthermore, a study of 27 ML lesions of the knee that movement exercises involving knee flexion, compression and vasopneumatic cryotherapy resolved the lesions 52% of the time while the remaining lesions required aspiration.^6^ For lesions which do not improve with initial conservative therapy, surgical management is also an option with combined percutaneous drainage, sclerotherapy with a sclerosant such as doxycycline and compression bandaging being the least invasive. A recent study showed that this combined therapy led to resolution of symptoms in 11 of 16 patients in 4 weeks time.^7^ It is also possible to excise the lesion and close off the space with cutaneofascial suturing.^8^

Morel-Lavallée lesions are a potential consequence of traumatic, shearing soft tissue injuries. As they are most easily and reliably treated early in their course, physicians should maintain an index of suspicion in the evaluation of the patient with persistent posttraumatic soft tissue swelling. While magnetic resonance imaging remains the radiological standard, ultrasonography provides a less costly but efficient alternative. The patient in this case underwent operative drainage of the ML lesion with closed-suction drain placement and postoperative compression therapy.

## Figures and Tables

**Figure 1:**
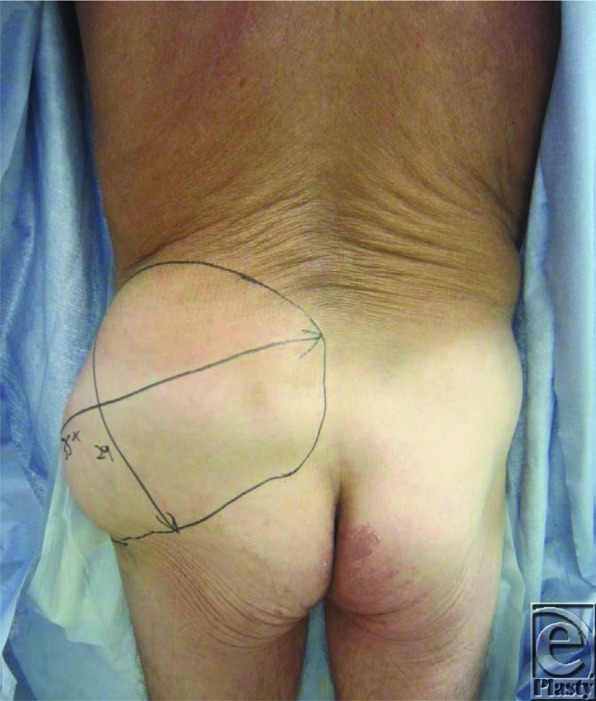
Posterior view demonstrating extensive fluid collection of left trochanteric/lower back region.

**Figure 2:**
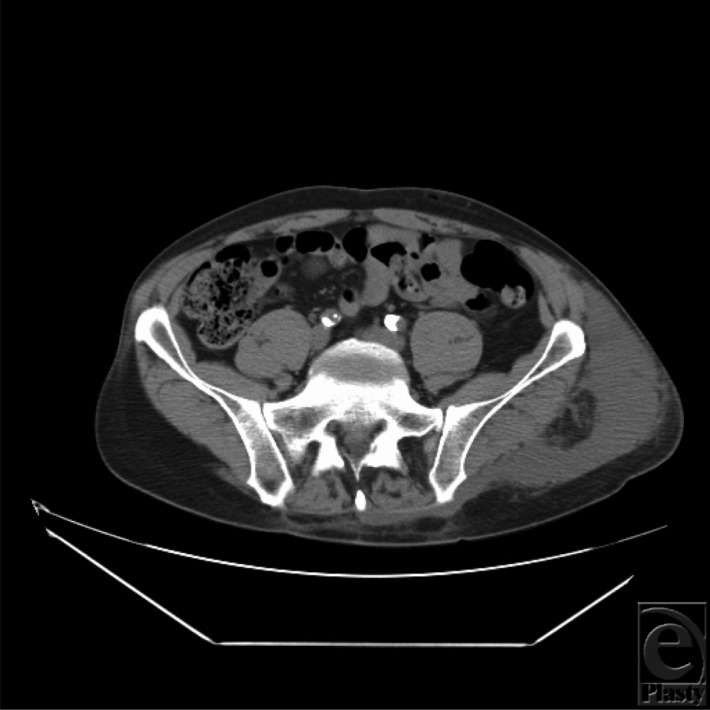
Magnetic resonance imaging axial image of the Morel-Lavallée lesion over the left trochanteric region with extension to the back.

**Figure 3:**
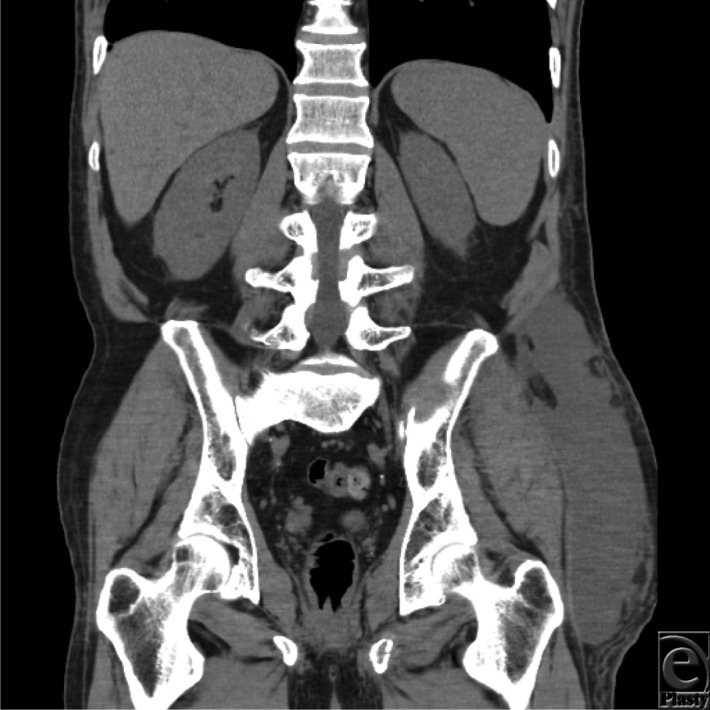
Magnetic resonance imaging coronal image of the Morel-Lavallée lesion in between the subcutaneous fat and the underlying fascia.
